# Metaheuristic-driven machine learning study for early detection and classification of Parkinson’s disease using feature prioritization with pelican optimization algorithm

**DOI:** 10.1097/MD.0000000000048699

**Published:** 2026-06-26

**Authors:** Proloy Kumar Mondal, Haewon Byeon

**Affiliations:** a Institute of Digital Anti-Aging Healthcare, Inje University, Gimhae-si, Republic of Korea; b Department of Future Technology, Korea University of Technology and Education, Cheonan, South Korea.

**Keywords:** explainable AI, feature selection, LightGBM, machine learning, Metaheuristic optimization, Pelican optimization algorithm

## Abstract

Parkinson disease (PD) is a degenerative disorder of the brain and afflicts approximately 6 in 10 people aged 50 years or older. PD patients have motor and speech problems, so regular visits to and monitoring of the patients are hard. It is necessary to detect the presence of PD promptly and accurately, since early treatment will contribute greatly to enhancing patients’ lives. As the number of aging people increases, there is a great demand for noninvasive, reliable, and remote diagnosis. In the current work, we studied 31 patients with PD and healthy subjects, their voice recordings, to create an automatic classification system. A Light Gradient Boosting Machine (LightGBM) classifier was adapted and boosted using metaheuristic-based feature selection (FS), namely the Pelican Optimization Algorithm (PAO). Hyperparameter optimization was made to optimize predictive performance. The models have been assessed on typical classification measures, i.e., accuracy, sensitivity, specificity, precision, and AUC. We classified using the baseline LightGBM classifier, with an accuracy of 95%. The resulting model had a better prediction accuracy of 97% after using PAO-based FS and hyperparameter optimization. More than that, the model was also sensitive, specific, precise, and had a high area under the curve, which validates its effectiveness at classifying PD. The paper shows that FS and hyperparameter tuning are effective approaches when applied to voice data and combined with LightGBM to detect PD as early as possible. The results point to the promise of noninvasive diagnostic systems based on the use of telemedicine to allow early intervention and enhance the lives of people with PD.

## 1. Introduction

Parkinson disease (PD) is a common neurological disorder that affects muscle movements and can impact the voice. It impairs mobility, speech, and posture, resulting in tremors, muscle stiffness, and bradykinesia.^[1]^ This condition is caused by the death of neurons, which reduces the level of dopamine in the brain. Low levels of dopamine disrupt communication at synapses, resulting in impaired motor function.^[2]^ Although the progression of symptoms may vary from patient to patient, imbalance and tremors appear to be the most common side effects of dopaminergic neuron death. Unfortunately, there is no cure for PD, so people with Parkinson (PWP) must rely on early detection and appropriate treatment to slow the progression of the disease.

Dopamine gives the body the ability to move correctly and smoothly. When 60% to 80% of dopamine-producing cells are destroyed, not enough dopamine is produced, and the motor symptoms of PD manifest. Early signs of PD appear in the enteric nervous system, lower brain stem, and olfactory tract. PD then spreads from these regions to the upper part of the brain, particularly the substantia nigra and cerebral cortex.^[3]^ The disease is thought to begin long before motor symptoms appear, and early symptoms include reduced or lost sense of smell, sleep disturbances, and constipation, which later progress to tremors and slow movements. Researchers are therefore looking for ways to detect these non-motor symptoms, which appear in the early stages of the disease, so that PD progression can be prevented.

The progression of PD is divided into 5 stages, and 90% of people with Parkinson disease (PWP) exhibit symptoms of vocal cord injury at stage zero. In addition to being easy to quantify, voice-related problems also fall into the realm of telemedicine or remote treatment.^[4]^ This eliminates the need for patients to physically visit the doctor; They can complete a simple test at home by recording audio over the phone. Common voice problems include dysphonia^[4]^ and dysarthria.^[5]^ Patients may be asked to hold a tone of a certain pitch for a longer period of time, called “sustained articulation,” or voice impairment may be assessed with a continuous speech test. These speech tests can be used to diagnose early stages of PD.

After early detection, doctors can restart treatment using therapeutic solutions or deep brain stimulation,^[6]^ which stimulate dopamine production and build neurons in the brain, thereby slowing the progression of PD. Due to the complex nature of this disease, there is no permanent cure so far. However, with early detection and proper medication, it is possible to control symptoms such as tremors and balance, helping patients return to a normal life.

## 2. Methods

### 2.1. Literature review

Previously, researchers have tried to predict the course of PD using genetic information, motion analysis and MRI scans. However, there has been very little research on early detection of hearing impairment. For example, Bilal et al^[7]^ used a support vector machine (SVM) model analyzing genetic data to predict the onset of PD in elderly individuals. With the SVM model they achieved a training accuracy of 0.889, while the improved SVM model reached 0.9183. In the case of PD, these results show that classification of auditory information is superior to genetic information.

To estimate the level of PD progression in the elderly, Thoser and Rane (2018) trained a random forest classifier, where they used keystroke data from the UCI’s telemonitoring dataset. Cordella et al^[8]^ classified patients with PD using auditory data in their study, although their models were highly dependent on MATLAB. Our research relies on freely-available models built in Python, known for their speed and low memory usage.

Most studies, such as Ali et al,^[9]^ have focused on integrating deep learning models into the analysis of phonation data, which helps predict when PD may manifest. This study highlights the applicability of deep learning methods in detecting PD. However, by failing to select the right relevant features, previous studies have missed opportunities to significantly improve the performance of deep neural networks (NNs).

To identify 7 major speech modes associated with PD, one study analyzed 22 variables using principal component analysis. Huang et al^[10]^ developed a conventional decision tree with 12 complex linguistic features derived from the MDVR-KCL^[11]^ dataset, which reduces the reliance on wearable devices for PD diagnosis. Due to the complexity of audio frequencies, instead of training Resnet models, Ozinski, M., Skalski et al^[12]^ decided to use images of audio data.

To reduce the influence of subjective assessment by physicians, Wroge et al^[13]^ used an unbiased machine learning (ML) approach to predict PD. However, they achieved a maximum accuracy of 85%, indicating that there is room for further improvement.

One study tested 4 classifiers decision tree, regression, deep neural, and NN for detecting PD.^[1]^

According to the statistics, the NN algorithm achieved the highest accuracy of 92.9 percent. Researchers in the cited reference^[14]^ identified freezing of gait (FOG) as a reliable indicator of sudden disability of movement in people with PD.

Empirical evidence shows that LSTM algorithm outperforms SVM in freezing of gait detection. Regular and close monitoring is crucial to detect the early stages of Parkinson, known as the prodromal or premotor stage.^[15,16]^

Wang et al^[17]^ used an in-depth approach to distinguish between asymptomatic and diagnosed patients with PD. They used twelve different ML models for dataset classification analyzing 401 voice biomarkers. A specially developed deep learning model, DEEP, demonstrated a classification accuracy of 96.45 percent. However, this has become financially unviable due to high memory requirements. Alkhatib et al^[18]^ differentiated between tremor and random motion in patients with PD using a linear classification method and achieved 95 percent accuracy. The researchers mainly monitored the patients’ movements and recommended that future studies include audio and sleep data. Using spatio-temporal analysis methods, Ricciardi et al^[19]^ examined brain MRI scans. To detect mild cognitive impairment, they used K-nearest neighbor, random forest, and decision tree models, which have been applied to patients with PD.

Haque, Amin Ul, et al^[20]^ used L1-support SVM on vocal datasets for patients with neurological disorders in their study. It is noteworthy that feature detection was not included in this study. The study by Aditi Govindu and colleagues, published in Procedia Computer Science 218 (2023) 249–261, focused on people aged 46 to 85 years. However, the study, described in Procedia Computer Science 00 (2019) 000-000, did not include young, healthy individuals.

As clinicians’ personal assessment may fail to detect subtle non-motor symptoms of PD, Mei et al^[21]^ emphasized the need for ML in PD diagnosis. Their meta-analysis examined dataset characteristics, ML methods used, and outcomes of 209 research papers.

According to reference,^[1]^ a combination of regression, NN, DMneural and decision tree was identified as the most effective classifier for PD detection, with the NN method achieving the highest accuracy of 92.9 percent.

AI-based methods for the diagnosis of PD are increasing in popularity, as these methods can handle large datasets and achieve high reliability without limiting the variability of the data.^[14,22]^ FOG has been identified as a reliable predictor of fall risk in Parkinson patients, and the authors identified FOG using LSTM (long short-term memory) techniques. Evidence suggests that LSTM is more effective than SVM in FOG detection.

### 2.2. Methodology

In the proposed method, biomedical voice measurement datasets collected from 31 individuals, 23 of whom were diagnosed with PD, were used.^[23]^ Data is pre-processed, analyzed and visualized to deeply understand the characteristics of the data. The classification model LightGBM is trained on 75% of the data. Models are trained to classify audio data from PD or healthy individuals based on frequency variation. Performance of the models was tested on 25% of the data and evaluated based on sensitivity, accuracy, precision, confusion matrix^[24]^ and ROC-AUC scores. The steps of a generic process, which shows the collection of data from the Data World database, splitting the data into test and training sets, training the model with ML algorithms, and validating the results. Next, we selected features using meta-heuristic algorithms such as the Pelican Optimization Algorithm (POA).

Also, we proposed an improvement of the model by applying a modified objective function to optimize the hyperparameters of LightGBM. A model is created and trained and then its performance is evaluated. We identified the most dominant features using LIME and SHAP analysis.

#### 2.2.1. Dataset description

**Figure 1.**
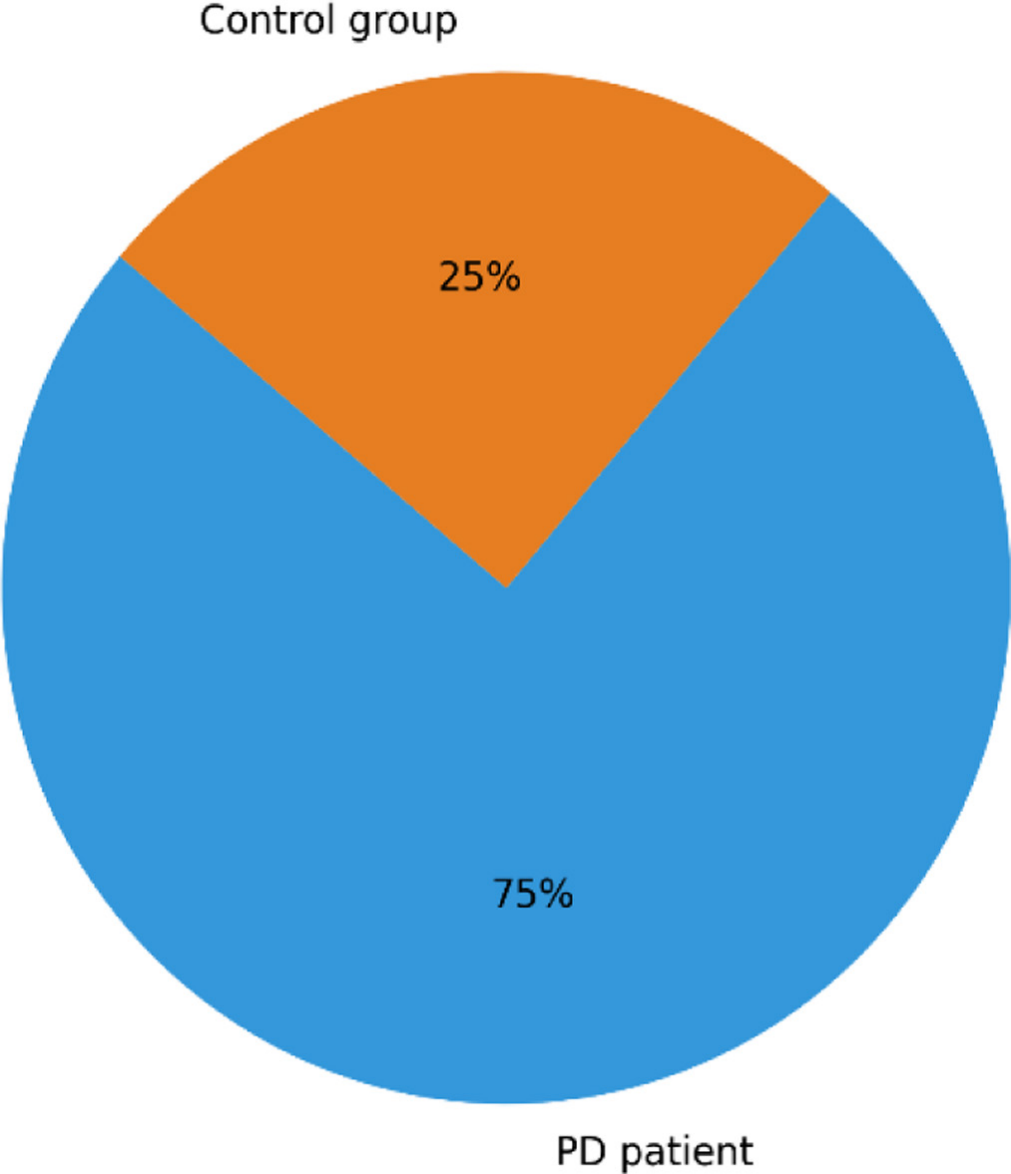
Class distribution of our dataset.

The dataset used in this study consists of the speech signals of 31 individuals from the National Center for Voice and Speech, Denver, Colorado. This dataset was created by Max Little of the University of Oxford and has been donated to the UCI Machine Learning Repository.^[23]^ Of the 31 subjects, 23 had PD and 8 belonged to the control group. The dataset includes a total of 195 biomedical voice measurements. Table 1 shows the voice measures used in the experiment. The “status” column of the database determines the class, with 0 assigned to healthy and 1 to PD patients. The class distribution is displayed in (Fig. 1) with 48 healthy phonemes and 147 PD-related phonemes, collected from these 31 individuals Units.

**Table 1:** Description of variables.

Features No	Voice measures
1	MDVP:Fo(Hz)
2	MDVP:Fhi(Hz)
3	MDVP:Flo(Hz)
4	MDVP:Jitter(%)
5	MDVP:Jitter(Abs)
6	MDVP:RAP
7	MDVP:PPQ
8	Jitter:DDP
9	MDVP:Shimmer
10	MDVP:Shimmer(dB)
11	Shimmer:APQ3
12	Shimmer:APQ5
13	MDVP:APQ
14	Shimmer:DDA
15	NHR
16	HNR
17	RPDE
18	D2
19	DFA
20	spread1
21	spread2
22	PPE
23	status

In Figure 1, a pie chart shows the breakdown of the study participants in terms of patients with Parkinson disease (PD) versus the control group. The blue segment, which represents PD patients, makes up 75 percent of the total participants, thus showing that 3/4 of the study subjects are diagnosed with Parkinson disease. On the other hand, the orange section denotes the control group, which constitutes a quarter of the sample, corresponding to the percentage of healthy people or individuals without Parkinson disease. This statistic indicates a larger number of PD patients than the control group in the data set.

A pie chart shows the breakdown of the study participants in terms of patients with PD versus the control group. The blue segment, which represents PD patients, makes up 75 percent of the total participants, thus showing that 3/4 of the study subjects are diagnosed with PD. On the other hand, the orange section denotes the control group, which constitutes a quarter of the sample, corresponding to the percentage of healthy people or individuals without PD. This statistic indicates a larger number of PD patients than the control group in the data set.

#### 2.2.2. Data preprocessing

**Figure 2.**
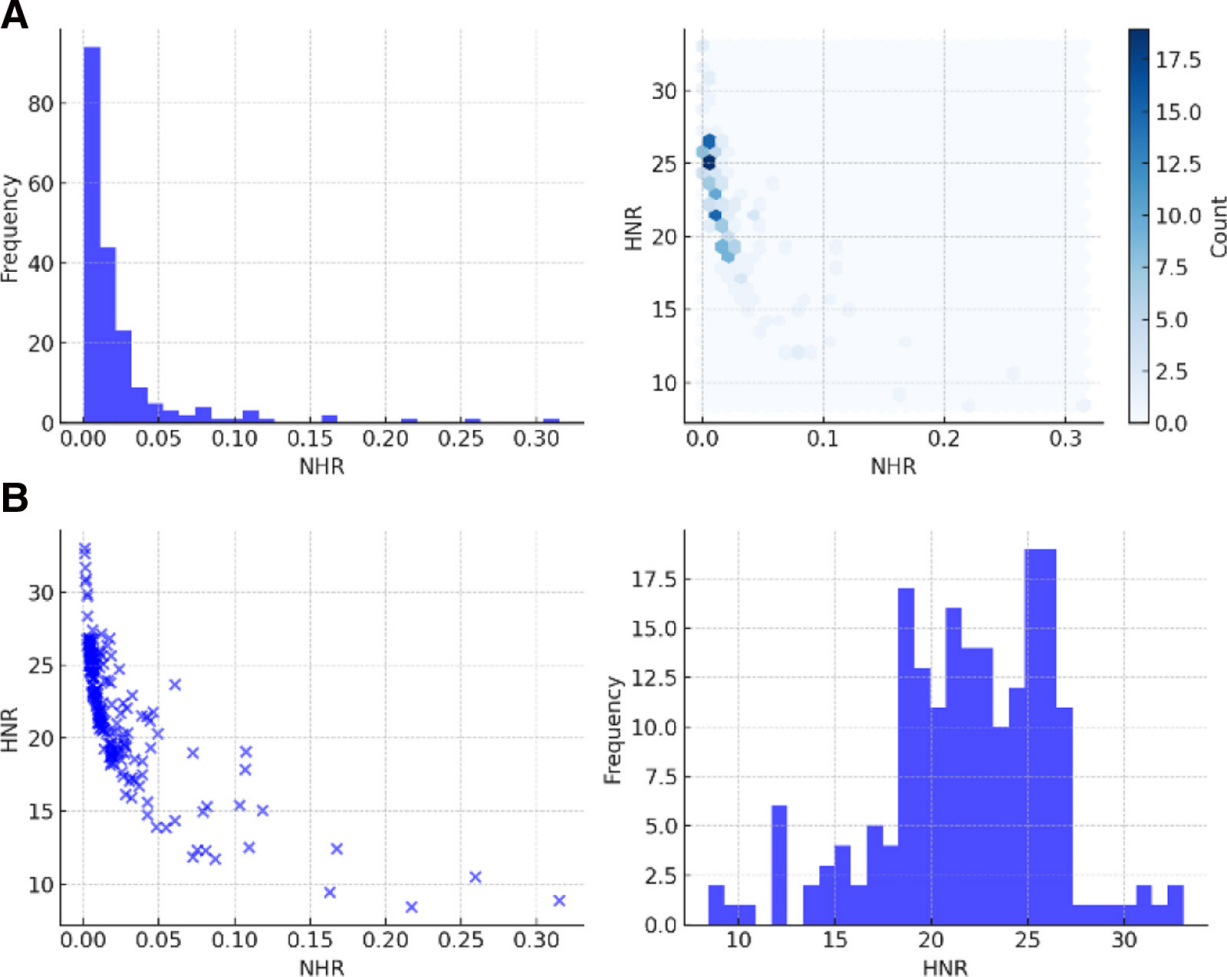
(a) NHR plot; (b) HNR plot.

(Fig. 2) presents an in-depth analysis of the relationship between noise-to-harmonics ratio (NHR) and harmonics-to-noise ratio (HNR), which provides valuable insight into voice characteristics. The histogram in (Fig. 2) (a) shows a concentration of NHR values close to 0.02, indicating low noise levels in most samples. The top-right hexbin plot (a) illustrates the density of NHR and HNR, where lower NHR values are often associated with higher HNR values, indicating that voice quality is more harmonious with less noise. The bottom-left scatter plot (b) further illustrates this relationship, where high HNR values correspond to low NHR values, indicating a clearer and less interfering voice signal. The bottom-right plot (b) shows the distribution of HNR, with most samples having values between 15 and 25, which generally indicates good harmonic quality in vocal recordings. Together, these visualizations show how vocal measures such as NHR and HNR can be used to distinguish people with PD from healthy people.

Figure 2 presents an in-depth analysis of the relationship between noise-to-harmonics ratio (NHR) and harmonics-to-noise ratio (HNR), which provides valuable insight into voice characteristics. The histogram in Figure 2 (a) shows a concentration of NHR values close to 0.02, indicating low noise levels in most samples. The top-right hexbin plot (a) illustrates the density of NHR and HNR, where lower NHR values are often associated with higher HNR values, indicating that voice quality is more harmonious with less noise. The bottom-left scatter plot (b) further illustrates this relationship, where high HNR values correspond to low NHR values, indicating a clearer and less interfering voice signal. The bottom-right plot (b) shows the distribution of HNR, with most samples having values between 15 and 25, which generally indicates good harmonic quality in vocal recordings. Together, these visualizations show how vocal measures such as NHR and HNR can be used to distinguish people with Parkinson disease from healthy people.

**Figure 3.**
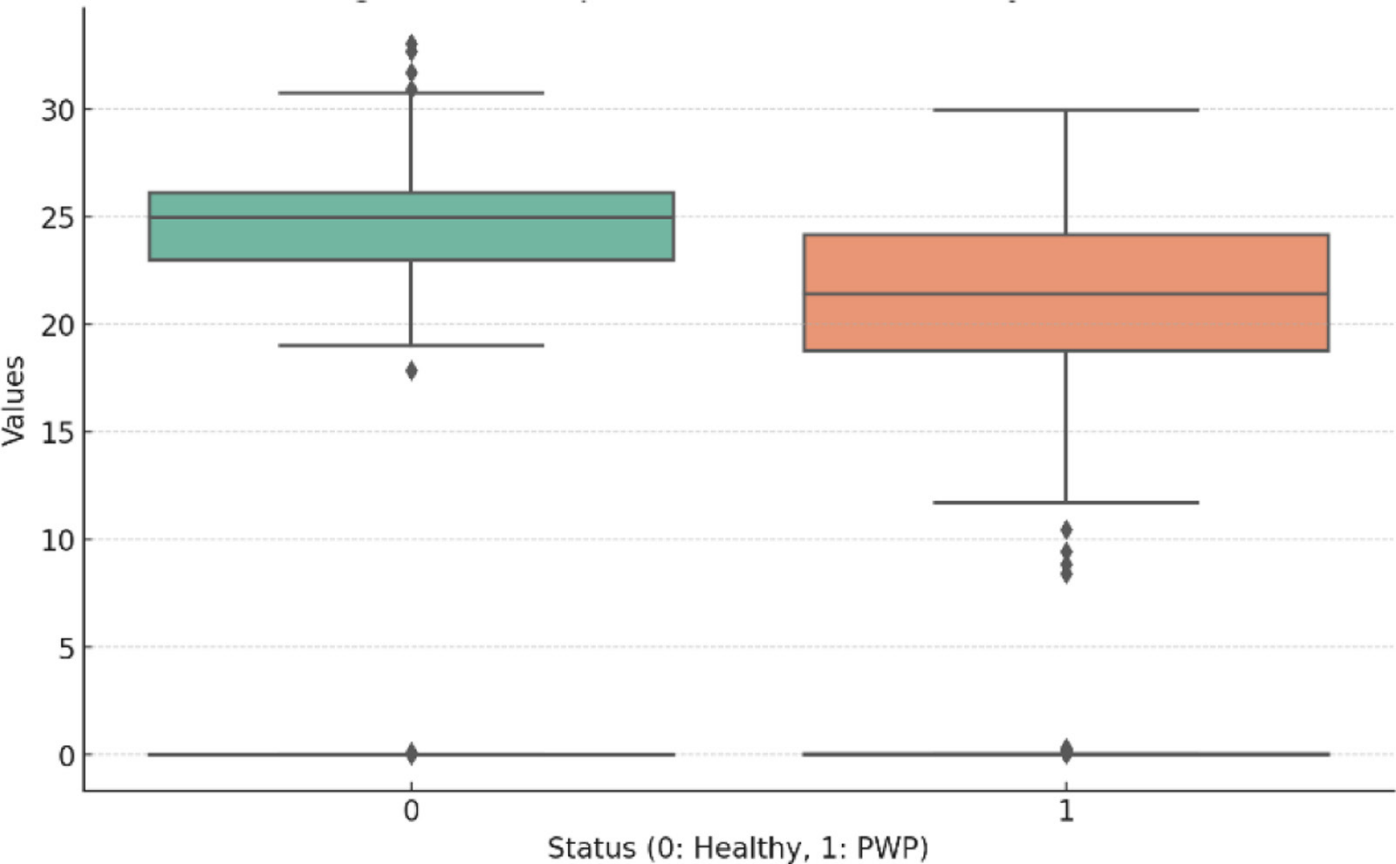
Box plot of NHR and HNR.

Figure 3 presents a box plot showing the comparison of NHR and HNR for healthy subjects (status = 0) and subjects with PD (status = 1). For people with Parkinson, the NHR values show more outliers, indicating an increase in the level of noise in their speech, whereas the distribution of healthy people is relatively more integrated. Similarly, HNR values are higher in healthy individuals, indicating better harmonic quality of their voices, but HNR values are lower and more varied in Parkinson patients, resulting in more outlier values. This box plot effectively shows how voice characteristics differ between the 2 groups, which can be helpful in diagnosing PD.

Figure 3 presents a box plot showing the comparison of noise-to-harmonics ratio (NHR) and harmonics-to-noise ratio (HNR) for healthy subjects (status = 0) and subjects with Parkinson disease (status = 1). For people with Parkinson, the NHR values show more outliers, indicating an increase in the level of noise in their speech, whereas the distribution of healthy people is relatively more integrated. Similarly, HNR values are higher in healthy individuals, indicating better harmonic quality of their voices, but HNR values are lower and more varied in Parkinson patients, resulting in more outlier values. This box plot effectively shows how voice characteristics differ between the 2 groups, which can be helpful in diagnosing Parkinson disease.

**Figure 4.**
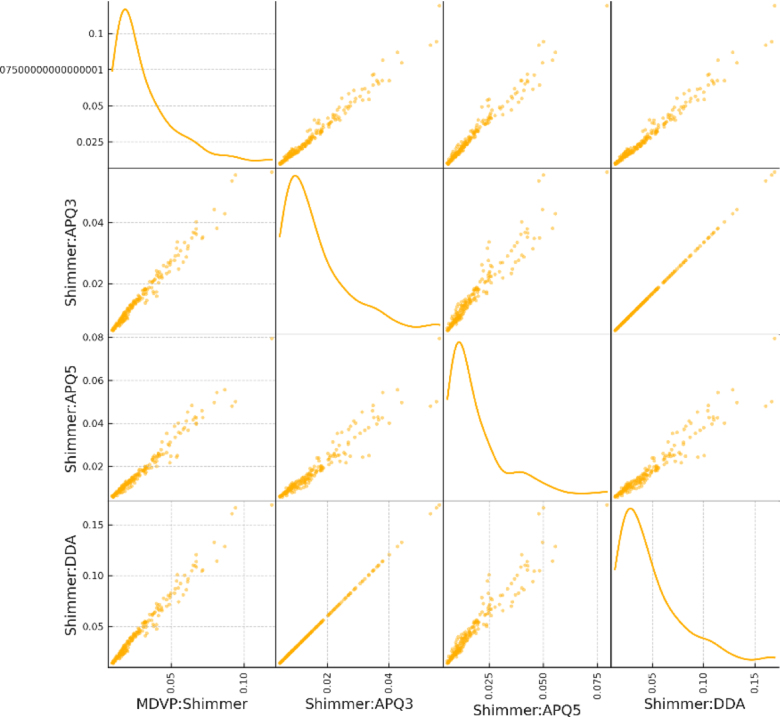
Pair plot of shimmer data.

The (Fig. 4) presents the relationships between various shimmer-related voice features from the PD dataset, specifically MDVP Shimmer, Shimmer, and Shimmer. The scatter plots reveal strong positive correlations between these features, indicating that as one shimmer measurement increases, others tend to increase as well. This suggests they capture similar aspects of voice amplitude variation. The KDE plots on the diagonal illustrate that most patients have lower shimmer values, with a few showing higher variations. These shimmer features are key in identifying amplitude irregularities in speech, which are often associated with PD.

Figure 4 presents the relationships between various shimmer-related voice features from the Parkinson disease dataset, specifically MDVP Shimmer, Shimmer, and Shimmer. The scatter plots reveal strong positive correlations between these features, indicating that as one shimmer measurement increases, others tend to increase as well. This suggests they capture similar aspects of voice amplitude variation. The KDE plots on the diagonal illustrate that most patients have lower shimmer values, with a few showing higher variations. These shimmer features are key in identifying amplitude irregularities in speech, which are often associated with Parkinson disease.

However, this section properly prepared the dataset with the selected independent variables and the target variable (status) to be loaded into the prediction model. We removed all noisy data from the converted CSV file. Also, in some instances the value of the independent variable was missing. Two resampling techniques are used to solve this problem: over-sampling and under-sampling. The over-sampling method ensures a more balanced representation of the minority class than the majority class by increasing the number of samples, which makes this method advantageous. We randomly split the dataset into 2 parts: 75% training set and 25% testing set. Later, the prepared training dataset was used as input to a ML model, which was intended to predict PD.

#### 2.2.3. Data classification with light

The LightGBM, or Light Gradient Boosting Machine, is a modern ML framework that has gained significant popularity due to its speed, efficiency and scalability.^[25]^ This framework, developed by Microsoft, is based on the principle of gradient boosting for fast and accurate processing of large datasets. One of the reasons behind its success is its ability to efficiently work with high-dimensional data, making it ideal for complex real-world tasks.

Compared to conventional decision tree-based models, LightGBM uses a leaf-based growth method, where the leaf with the highest profit is grown first. This technique helps to achieve better accuracy, reducing the cost of computation. In addition, it provides native support for categorical features, thereby reducing the need for data preprocessing and simplifying the workflow for data scientists.

**Figure 5.**
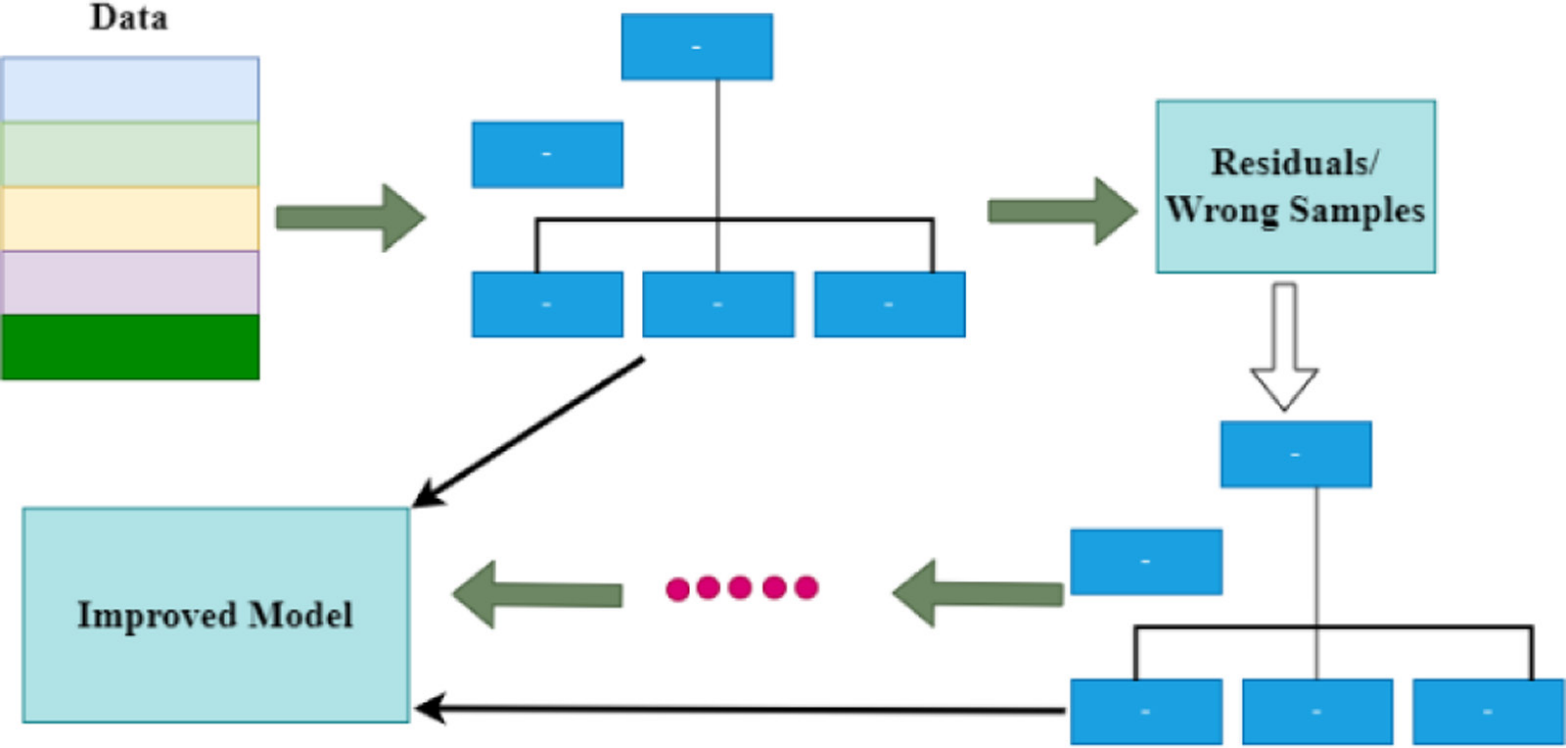
Schematic representation of the LightGBM classification algorithm.

LightGBM performance is further enhanced by its parallel and GPU-based training support, which enables it to process large datasets quickly and efficiently. This makes it a popular choice due to its speed and accuracy for tasks such as regression, classification and ranking. Figure 5 illustrates the complete process of classification utilizing the LightGBM algorithm.

The LightGBM, or Light Gradient Boosting Machine, is a modern machine learning framework that has gained significant popularity due to its speed, efficiency and scalability.^[25]^ This framework, developed by Microsoft, is based on the principle of gradient boosting for fast and accurate processing of large datasets. One of the reasons behind its success is its ability to efficiently work with high-dimensional data, making it ideal for complex real-world tasks.

Compared to conventional decision tree-based models, LightGBM uses a leaf-based growth method, where the leaf with the highest profit is grown first. This technique helps to achieve better accuracy, reducing the cost of computation. In addition, it provides native support for categorical features, thereby reducing the need for data preprocessing and simplifying the workflow for data scientists.

LightGBM performance is further enhanced by its parallel and GPU-based training support, which enables it to process large datasets quickly and efficiently. This makes it a popular choice due to its speed and accuracy for tasks such as regression, classification and ranking. Figure 5 illustrates the complete process of classification utilizing the LightGBM algorithm.

#### 2.2.4. Feature selection

Feature selection (FS) is a preprocessing step in data science, where key features of the problem are identified.^[26]^ Accurate determination of features has a major impact on increasing classification accuracy. The overall performance of ML methods can be improved by reducing dimensionality.^[27]^ Jain and Singh noted, “The application of classification algorithms to disease datasets has yielded positive results in the development of useful, automated and intelligent diagnostic systems for chronic diseases.”^[27]^

FS has many advantages, such as saving time for future data collection, understanding the cause of disease, low computational cost, and no performance degradation.^[26]^ In this study, we used FS to diagnose PD. Various FS methods have been tested, including univariate selection (US) and feature importance (FI). The FI method is intended for use in classification algorithms, and we used it in the LightGBM classification method. These FS methods are selected based on the performance of the classifier. The FI method assigns a score to each attribute, where a higher score means the attribute is more relevant to the output variable. In the RFE method, features that fit a model are progressively removed until the desired number of features is met. The features are ranked and removed through an iterative loop.

Identifying the optimal feature set is a challenging task,^[28]^ which is often solved using metaheuristic algorithms.^[29]^ Due to its ability to efficiently navigate complex search spaces and yield good results with low computational effort, FS is increasingly using metaheuristic algorithms.^[30]^ In this study, Comprehensive Learning POA, a well-known swarm-based metaheuristic method, is adopted for FS.

#### 2.2.5. POA

The POA is inspired by nature, specifically the hunting strategy of pelicans.^[31]^ These birds’ methods of diving for prey and surfacing for fishing serve as the basis for the algorithm’s design and functionality. POA is conducted in 2 main stages. The first is the exploration phase, where pelicans (as search agents) explore the search space. A new position is adopted only if it improves the value of the objective function, which helps prevent entry into the non-optimal region. This process is modeled mathematically for efficient exploration. The second phase, the exploitation phase, simulates pelicans flapping their wings at the surface of the water to lift fish, which increases the local search capabilities of the algorithm and helps it arrive at better solutions by examining nearby points. The motion and decision-making processes of pelicans while searching for prey have been mathematically modeled. The performance of POA is evaluated through simulation of 23 objective functions, both unimodal and multimodal. The results show that POA outperforms 8 well-known optimization algorithms, such as particle swarm optimization (PSO) and genetic algorithm (GA), by maintaining a strong balance between exploration and exploitation. Beyond theory, POA has also been applied to real-world engineering design problems, proving its practical effectiveness. In summary, POA combines biological inspiration and mathematical modeling to create a powerful optimization tool, which strikes an effective balance between exploration and exploitation to find optimal solutions.

**Table:** 

**Algorithm 1:** Feature selection using POA
Initialize	
Compute the POA population size (N) and the number of rounds (T) Initializing pelican’s location and compute the fitness value.	
For t = 1 to T	
	Create prey’s place randomly
	For i = 1 to N Phase 1: Moving in the direction of prey, For j = 1 to m Evaluate the newly attained status of the jth dimension End. Update the ith population member Phase 2: Winging on the surface of the water For j = 1 to m Evaluate the newly attained position of the jth dimension End Update the ith population member. End. Update optimum candidate solutions.
	End Display Optimal candidate solutions using POA Stop POA

#### 2.2.6. Model evaluation

We applied different model evaluation criteria, especially accuracy score, F1 score and sensitivity score. These metrics are calculated based on the confusion matrix to measure the performance of the proposed model. Higher values of scores indicate that the model performed better than other ML models. The confusion matrix provides values for true positives (TP), false positives (FP), true negatives (TN), and false negatives (FN), which are important in determining model performance. Here, TP indicates correct prediction of PD, FP indicates incorrect prediction of PD, TN indicates correct prediction of non-PD and FN indicates incorrect prediction of non-PD. The evaluation metrics are calculated using Equations (1) to (4).


$$\begin{array}{c} Accuracy=\frac{TP+TN}{TP+FP+TN+FN} \end{array}$$ (1)

$$\begin{array}{c} F1\;Score=2\times \frac{precision\times reca}{precision+reca} \end{array}$$ (2)

$$\begin{array}{c} Here,\;\;precision=\frac{TP}{TP+FP} \end{array}$$ (3)

$$\begin{array}{c} reca=\frac{TP}{TP+FN} \end{array}$$ (4)

In addition to a classification model, we also used other performance evaluation methods, which are described in the next section. A receiver operating characteristic (ROC) curve is a graphical representation that depicts the classifier performance of a ML model at different possible thresholds. In the ROC curve, the vertical axis (𝑦-axis) represents the true positive rate (TPR) or sensitivity, and the horizontal axis (𝑥-axis) represents the false positive rate (FPR) or specificity. Also, the area under the receiver operating characteristic curve (AUC) is calculated, which is based on the measurement of the area covered by the ROC curve. Higher values of AUC indicate better performance of the model.

#### 2.2.7. Machine learning classifier and XAI techniques

This study focuses on selecting an optimal ML model for PD classification using the labeled dataset, emphasizing the need for XAI to provide accurate, robust, and understandable explanations of model predictions and behaviors.^[32]^ The dataset is used for both training and testing the ML model. As discussed in the previous section, all features are used as inputs for the ML models for PD classifications. In this research, classification ML-based algorithms are assessed based on evaluation metrics, which include LightGBM.

The study incorporates XAI techniques to explain the decisions made by the ML models. Among the XAI methods explored are Shapley additive explanations (SHAP), which provide a global measure of the feature importance. Local Interpretable Model-agnostic Explanations (LIME) and SHAPASH offer detailed insights into the model’s reasoning in arriving at a prediction. The weights of the surrogate model help to calculate the SHAP values that define the importance of each input feature.

Eq. (5) is used to evaluate the SHAP value for individuals’ instances.


$$\begin{array}{c} \emptyset \left(x\right)=\underset{i}{\sum }\;\emptyset_{i}\left(x\right) \end{array}$$ (5)

Where ∅(x) denotes the overall SHAP value is implied for the example x and $\emptyset_{i}\left(x\right)$ the SHAP value for an attribute is presented, the importance of each attribute can be determined using the SHAP values in the example.^[33]^ SHAP standards are very useful, as they provide deep insight into the model and provide important information on the properties contributing to the prediction, making it transparent and valuable in clinical application. Waterfall plots are used for visual representation of SHAP values, demonstrating how each feature is contributing to specific predictions. On the other hand, bee or bar plots show the overall importance of features. Dependence plots illustrate the relationship between attributes and force plots help understand the influence of specific attributes. Collectively, these visual tools help to understand the model, solve problems, and improve the model.

LIME evaluates changes in predictions by changes in input data. This approach helps to understand the decision-making process of black box models and provides insight into how the models behave locally.^[34]^ Eq. (6) represents the mathematical expression of the LIME method for a specific example.


$$\begin{array}{c} \;\Psi \;\left(x\right)=\mathrm{\backslash arg}\underset{h\in H}{\min}\;ℒ\;\left(\;f,H,\overset{\prime }{\underset{x}{\pi }}\right)+\chi \left(h\right) \end{array}$$ (6)

The local kernel, denoted by $\overset{\prime }{\underset{x}{\pi }}$, is used for the loss calculation. ψ(𝑥) represents the interpretation. The unreliability of ℎ is measured by the inverse value of the local fidelity in the approximation L $ℒ\;\left(\;f,H,\overset{\prime }{\underset{x}{\pi }}\right)$, 𝜒(ℎ) expresses the complexity of the local model ℎ, which is also referred to as a penalty constraint to reduce the time complexity. Therefore, the optimization of local accuracy is crucial, i.e. reducing the lack of fidelity in the proposed ML model.

## 3. Results

The results of hyperparameter tuning are detailed in Section A on the experimental evaluation of our proposed LightGBM model. In addition, the relevance of the features of the XAI algorithm mentioned in Section B and the analysis results are also evaluated, which are discussed in Section C. In addition, the proposed model is compared with other conventional methods and comparative analysis with previous studies, which are discussed in detail in Sections D and E, respectively.

### 3.1. Performance analysis

**Figure 6.**
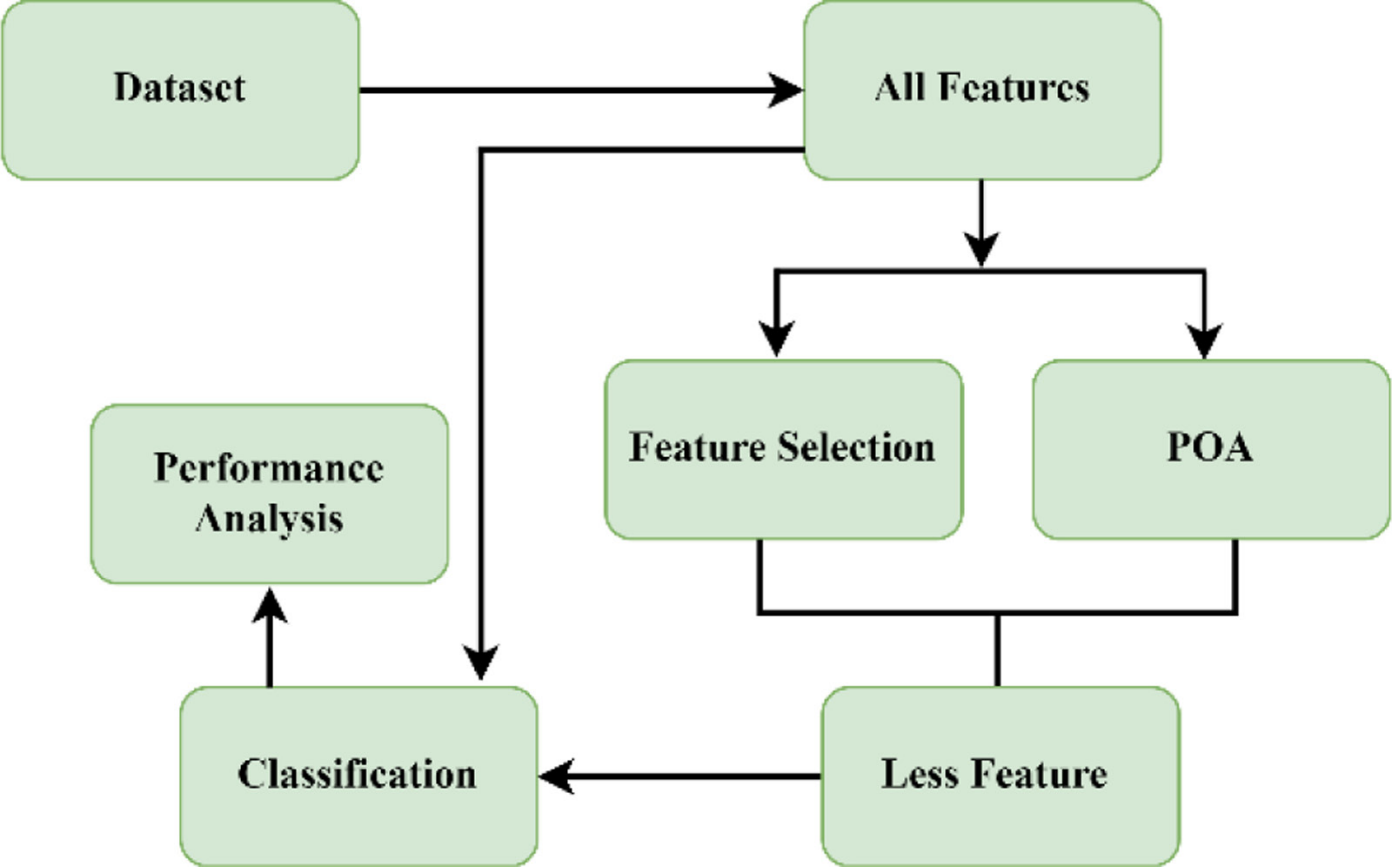
Overall methodology of this work.

We have successfully completed training our LightGBM classifier. Figure 6 provides an informative summary of the results analysis process performed in this thesis. It is clear from the diagram analysis that we used all 22 features of the processed dataset to train the model before and after hyperparameter optimization (HPO). Next, through the FS process, we proceed to train the optimized model using each feature. Finally, we examine the results in depth. Feature importance is determined using XAI, which further improves feature analysis.

Figure 6 provides an informative summary of the results analysis process performed in this thesis. It is clear from the diagram analysis that we used all 22 features of the processed dataset to train the model before and after hyperparameter optimization (HPO). Next, through the feature selection (FS) process, we proceed to train the optimized model using each feature. Finally, we examine the results in depth. Feature importance is determined using XAI, which further improves feature analysis.

### 3.2. Hyper parameter tuning outcome

To make the RF classifier more accurate and reliable, we selected the most suitable parameters for the hyperparameter (HP). The maximum depth, N estimator, learning rate and maximum features of LightGBM classifier have already been improved. As mentioned earlier, manual tuning process is very long and time consuming. We used 3 different strategies Grid Search CV (GS), Random Search CV (RS), and POA to achieve the optimal HP value.

Table 2 shows the performance of 3 different HPO algorithms, namely Random Search (RS), Grid Search (GS) and POA, evaluated by parameter tuning on our dataset. Average accuracy and various hyperparameters such as maximum depth, minimum sample split, learning rate and number of estimators for each model were used as the main metrics. Both Random Search (RS) and Grid Search (GS) achieved an average accuracy of 94.87%. However, there are differences in the hyperparameters of the models tuned by these 2 algorithms. For example, RS tuned the maximum depth to 11, used 20 as the minimum sample split, and used a learning rate of 0.05 with 250 estimators. On the other hand, GS achieved the same accuracy using a maximum depth of 5, a learning rate of 0.1, and 100 estimators. Both RS and GS showed almost the same performance through effective parameter adjustments. On the other hand, the POA, which is a metaheuristic method, showed a lower accuracy of 81.53%. It used a maximum depth of 15, a small sample split of 6, a learning rate of 0.25, and only 20 estimators. These parameter adjustments may lead to overfitting or underfitting, which may lead to a decrease in the performance of POA compared to RS and GS.

**Table 2:** HPO (hyperparameter hyperparameter optimization) algorithms outcome overview.

Algorithms	Mean accuracy	Max depth	Min sample split	Learning rate	N estimators
RS	94.87%	11	20	0.05	250
GS	94.87%	5	20	0.1	100
POA	81.53%	15	6	0.25	20

### 3.3. Performance of classification algorithm

**Figure 7.**
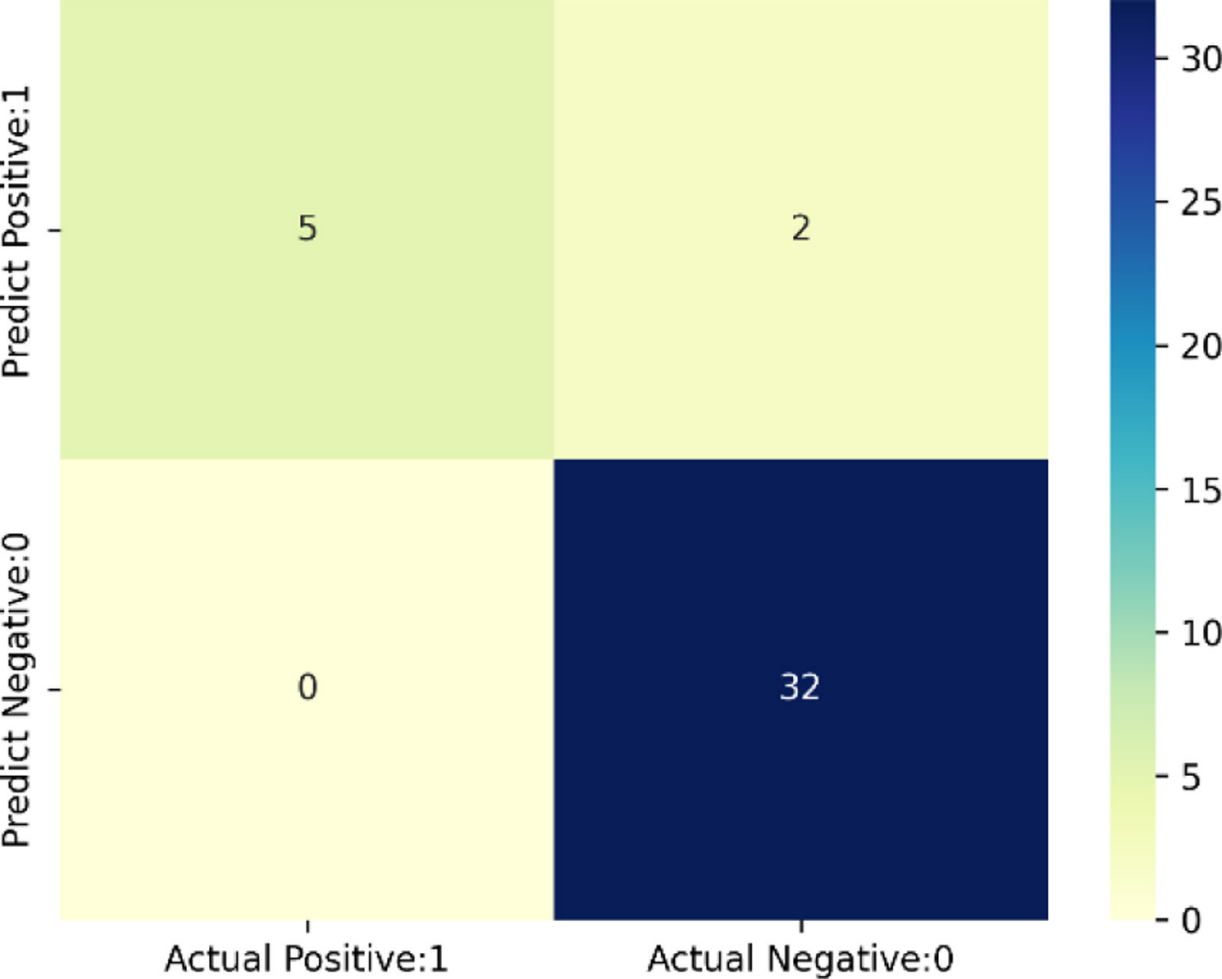
Confusion matrix for LightGBM classifier.

**Figure 8.**
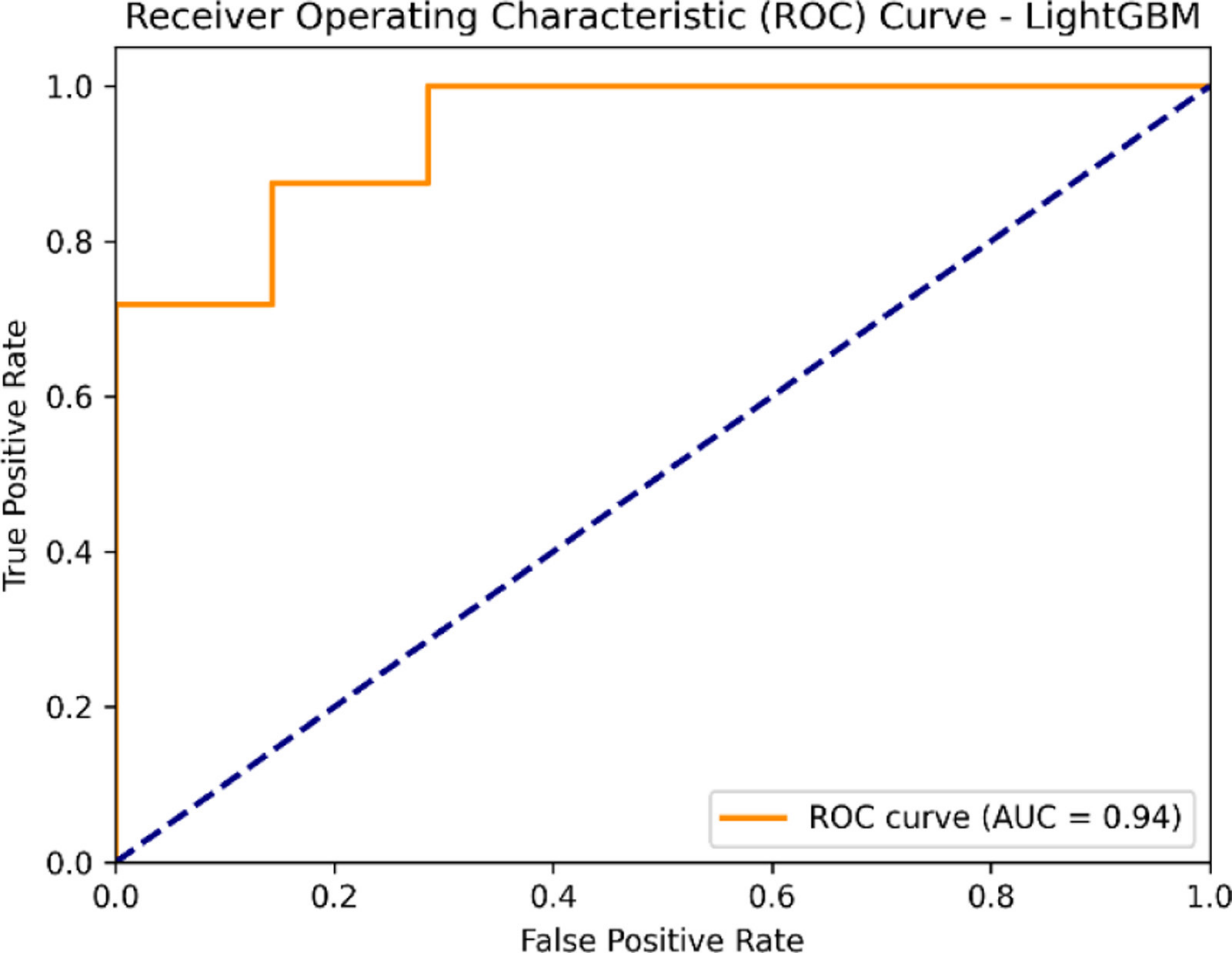
ROC curve 3 different classes for LightGBM.

The confusion matrix of the LightGBM model, shown in (Fig. 7) provides important insight into the classification performance of the model. It exhibits strong accuracy, especially in correctly identifying true positive and true negative events. The model successfully detected 32 true negatives and 5 true positives, and only 2 false positives occurred, with no false negatives detected. This accuracy shows that the LightGBM algorithm is capable of making highly efficient and reliable predictions for the dataset. (Fig. 8) shows the ROC curve of the LightGBM model, where the AUC is 0.94. This high AUC score indicates that the model is able to correctly distinguish positive and negative classes at different decision thresholds. The curve is far from the diagonal line, which represents random estimation, indicating that the model can maintain a high true positive rate while keeping the false positive rate low. This balance indicates the robustness of the overall classification performance of the model, which is helpful in achieving high predictive accuracy.

The confusion matrix of the LightGBM model, shown in Figure 7 provides important insight into the classification performance of the model. It exhibits strong accuracy, especially in correctly identifying true positive and true negative events. The model successfully detected 32 true negatives and 5 true positives, and only 2 false positives occurred, with no false negatives detected. This accuracy shows that the LightGBM algorithm is capable of making highly efficient and reliable predictions for the dataset.

Figure 8 shows the receiver operating characteristic (ROC) curve of the LightGBM model, where the AUC (area under the curve) is 0.94. This high AUC score indicates that the model is able to correctly distinguish positive and negative classes at different decision thresholds. The curve is far from the diagonal line, which represents random estimation, indicating that the model can maintain a high true positive rate while keeping the false positive rate low. This balance indicates the robustness of the overall classification performance of the model, which is helpful in achieving high predictive accuracy.

Although the accuracy of the LightGBM model is high, the lack of interpretability poses a challenge in clinical application. In order to increase the transparency and credibility of the model, especially in the healthcare field, the inclusion of interpretable AI (XAI) techniques is needed. This will allow clinicians to understand how input characteristics are influencing outcomes. As a result, the integration of interpretable AI models will increase their effectiveness in personalized treatment and clinical decision-making, and it will help gain more credibility and acceptance in areas such as healthcare.

**Figure 9.**
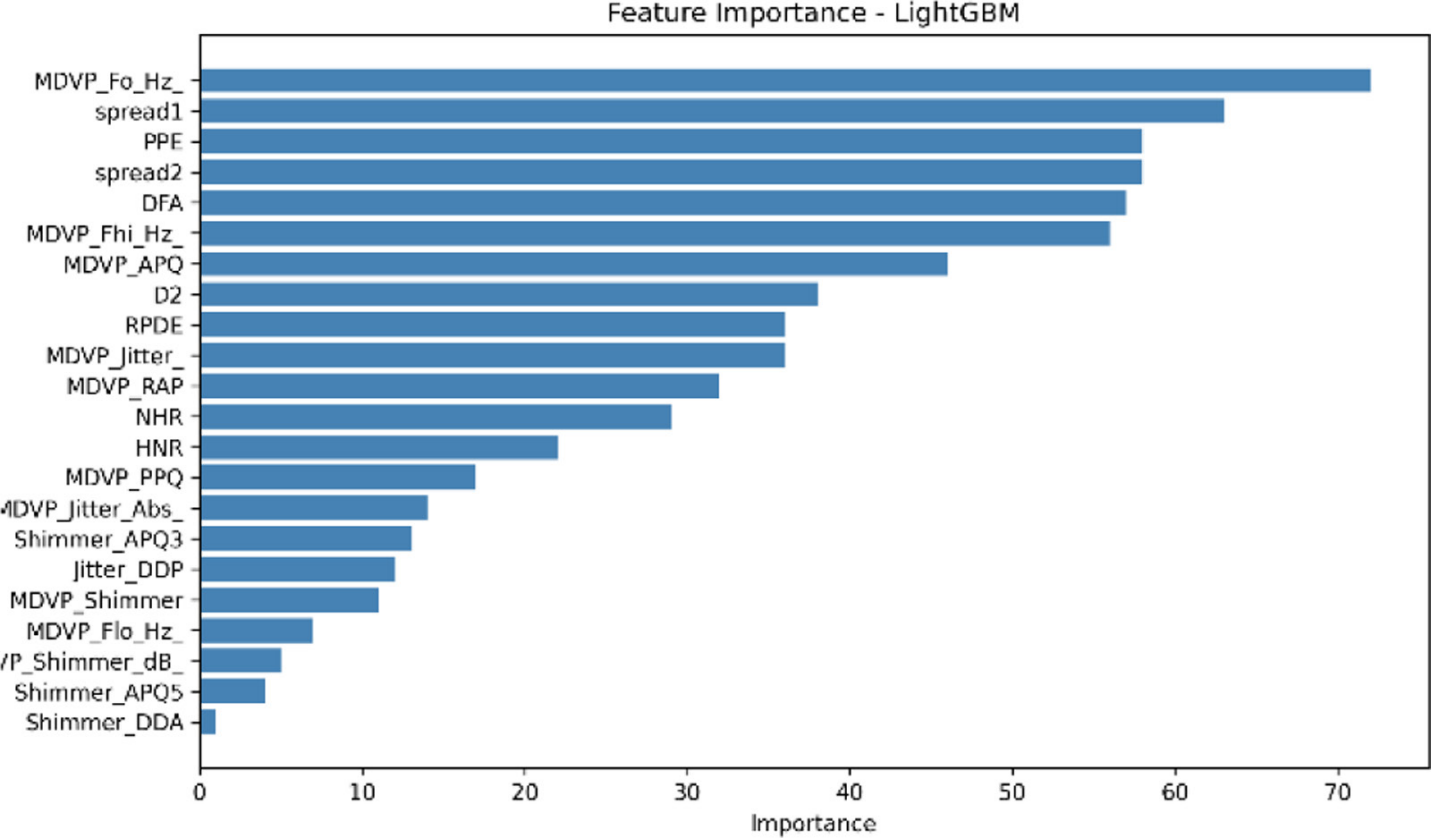
Features importance of the LightGBM for classification of PD disease.

With the aim of increasing transparency in AI-driven diagnostics, advanced XAI techniques, specifically SHAP and LIME, have been used in this study. The feature importance plot, displayed in (Fig. 9) highlights the main features the model contributes to the prediction of the dataset. Among these, features such as difference entropy, joint entropy and difference variance have the greatest impact on model results. The implications of these features show that they play an important role in diagnosis, indicating significant areas for further research in the future.

With the aim of increasing transparency in AI-driven diagnostics, advanced XAI techniques, specifically SHAP and LIME, have been used in this study. The feature importance plot, displayed in Figure 9 highlights the main features the model contributes to the prediction of the dataset. Among these, features such as difference entropy, joint entropy and difference variance have the greatest impact on model results. The implications of these features show that they play an important role in diagnosis, indicating significant areas for further research in the future.

LightGBM model’s 8 feature importance plots illustrate the relative importance of different features in the model’s predictions. Attribute importance is a key component of model interpretation, as it helps determine which variables have the greatest effect on outcomes. In this particular case, the most important feature is MDVP_Fo_Hz_, which refers to the fundamental frequency or pitch of the voice, and is considered an important indicator in speech-related tasks such as voice disorder detection. Next, features such as spread1, PPE, and spread2 are also ranked high, indicating their strong role in model decision-making. These features may represent different acoustic qualities, such as voice variations or speech irregularities. Further down the list are features like Shimmer_APQ5 and Shimmer_DDA, which contribute to the model, but to a lesser extent. By understanding these rankings, it is possible to further improve the model, focus on the most important inputs, and increase performance in voice analysis or related tasks.

LightGBM model’s 8 feature importance plots illustrate the relative importance of different features in the model’s predictions. Attribute importance is a key component of model interpretation, as it helps determine which variables have the greatest effect on outcomes. In this particular case, the most important feature is MDVP_Fo_Hz_, which refers to the fundamental frequency or pitch of the voice, and is considered an important indicator in speech-related tasks such as voice disorder detection. Next, features such as spread1, PPE, and spread2 are also ranked high, indicating their strong role in model decision-making. These features may represent different acoustic qualities, such as voice variations or speech irregularities. Further down the list are features like Shimmer_APQ5 and Shimmer_DDA, which contribute to the model, but to a lesser extent. By understanding these rankings, it is possible to further improve the model, focus on the most important inputs, and increase performance in voice analysis or related tasks.

Figure 11 represents a LIME (local interpretable model-agnostic interpretation) analysis, which provides an interpretation of the model’s predictions for a specific instance of PD diagnosis. According to the prediction probabilities, the model classified this instance as “Parkinson’s” with a probability of 0.99 and “Healthy” with a probability of only 0.01. The features shown on the right played an influential role in this decision. Features such as PPE, Spread1, and Jitter: DDP are shown with their values, and their contribution to PD prognosis is indicated by adjacent orange bars. For example, a PPE value of 0.21, a Spread1 value of -5.63, and a Jitter: DDP value of 0.01 serve as strong indicators of “Parkinson’s” classification. The length of the orange bar shows the intensity of the effects of the features. Model terms for PD diagnosis are displayed on the left. For example, the value of PPE must be less than or equal to 0.25 and the value of Spread1 must be less than or equal to -5.18 to predict PD. These thresholds indicate how well the features match the model’s learned rules. This LIME explanation helps explain how specific feature values in the model played a role in the diagnosis of PD, bringing clarity to the black-box nature of ML models.

**Figure 10.**
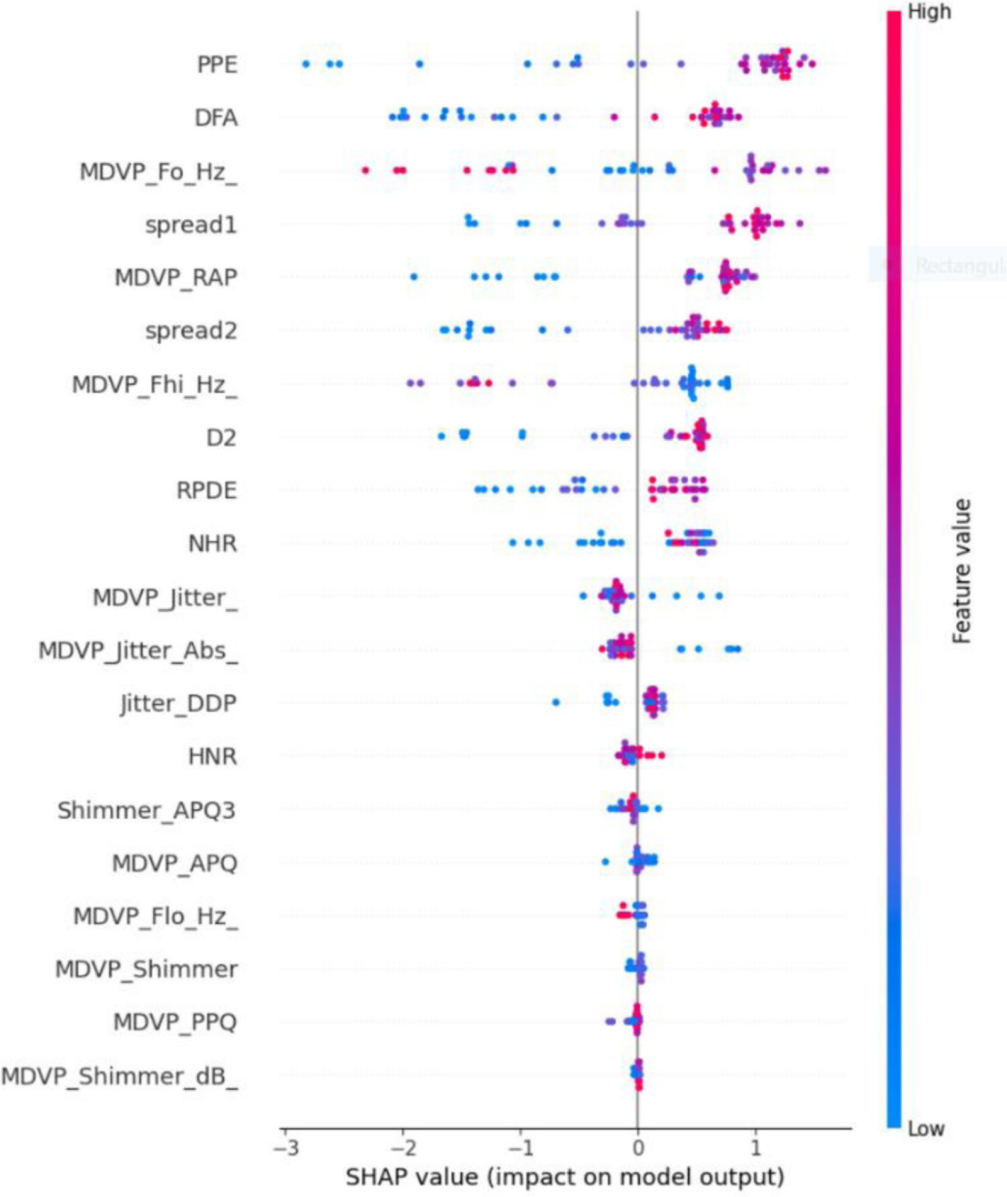
SHAP shows global and local feature importance and produce the values for every individual feature.

The SHAP analysis plot (Fig. 10) demonstrates the effect of various features on the model’s predictions. Each feature, such as PPE and DFA, is shown with SHAP values, indicating their effect on the model’s output. High SHAP values drive the prediction in one direction (e.g., towards a diagnosis of Parkinson disease), whereas low values influence the opposite direction. The color gradient represents the value of the feature, with blue indicating low and red high values. For example, the PPE attribute has a significant impact on prediction, where both high and low values of it affect the model’s decision. This analysis helps to understand how each feature contributes to the final prediction.

**Figure 11.**
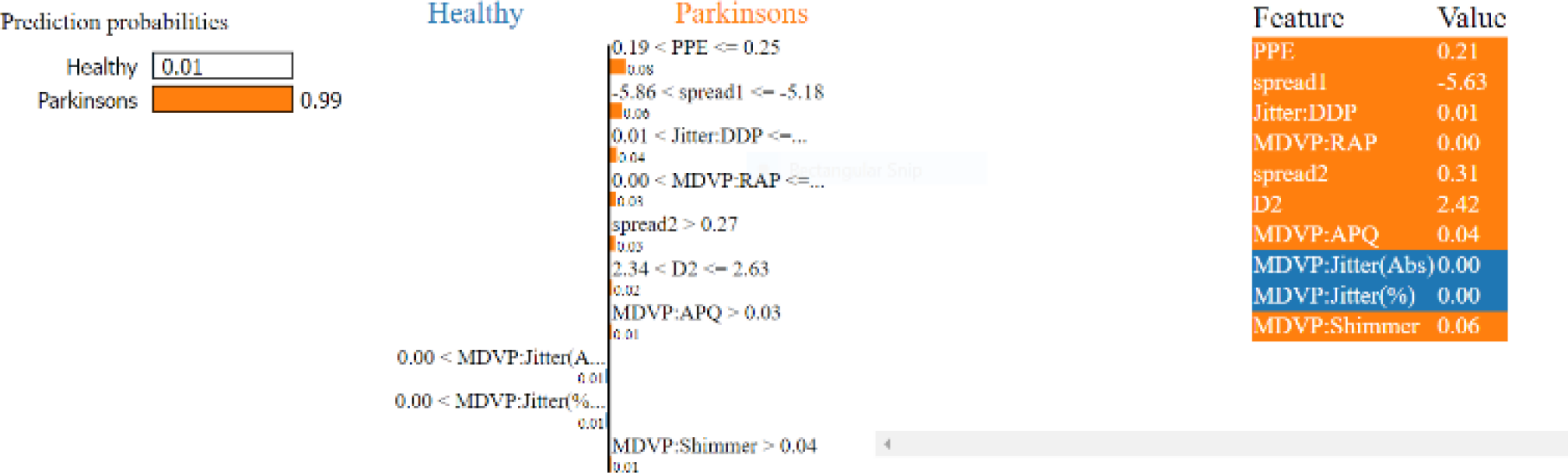
Lime shows analysis.

Figure 11 represents a LIME (local interpretable model-agnostic interpretation) analysis, which provides an interpretation of the model’s predictions for a specific instance of Parkinson disease diagnosis. According to the prediction probabilities, the model classified this instance as “Parkinson’s” with a probability of 0.99 and “Healthy” with a probability of only 0.01. The features shown on the right played an influential role in this decision. Features such as PPE, Spread1, and Jitter: DDP are shown with their values, and their contribution to Parkinson disease prognosis is indicated by adjacent orange bars. For example, a PPE value of 0.21, a Spread1 value of -5.63, and a Jitter: DDP value of 0.01 serve as strong indicators of “Parkinson’s” classification. The length of the orange bar shows the intensity of the effects of the features. Model terms for Parkinson disease diagnosis are displayed on the left. For example, the value of PPE must be less than or equal to 0.25 and the value of Spread1 must be less than or equal to -5.18 to predict Parkinson disease. These thresholds indicate how well the features match the model’s learned rules. This LIME explanation helps explain how specific feature values in the model played a role in the diagnosis of Parkinson disease, bringing clarity to the black-box nature of machine learning models.

The SHAP analysis plot (Fig. 10) demonstrates the effect of various features on the model’s predictions. Each feature, such as PPE and DFA, is shown with SHAP values, indicating their effect on the model’s output. High SHAP values drive the prediction in one direction (e.g., towards a diagnosis of PD), whereas low values influence the opposite direction. The color gradient represents the value of the feature, with blue indicating low and red high values. For example, the PPE attribute has a significant impact on prediction, where both high and low values of it affect the model’s decision. This analysis helps to understand how each feature contributes to the final prediction.

### 3.4. Features selection performance outcome

To improve the accuracy of the LightGBM classifier and reduce the data dimension, we implemented an FS algorithm. The FS process is accomplished using a meta-heuristic algorithm known as POA. The accuracy of the LightGBM classifier was evaluated and the fitness function was generated from that evaluation. Other performance metrics, such as F1 scores, precision, and recall, were not taken into account. We experimented with the “p_r” parameter of the POA algorithm in the range of 0.21, which indicates the learning rate potential. A value of 0.25 is determined as optimal for “p_r.” The values of the “pop_size” and “epoch” parameters are 20 and 10 respectively. The results for different “p_r” values which includes the set of features obtained from the POA process. Table 3 contains the sets of features that were the best features returned from the POA process.

**Table 3:** Features are generated based on POA.

Optimization Algorithm	Feature name	Accuracy
	MDVP:Fo(Hz)	
POA	spread1	97%
	RPDE	
	spread2	
	MDVP:Fhi(Hz)	

Using Metaheuristic Algorithm POA identified 5 important features, which were helpful in improving the performance of LightGBM classifier. These features are MDVP:Fo(Hz), spread1, RPDE, spread2, and MDVP:Fhi(Hz). At first, the accuracy of the LightGBM classifier was 95%, but after applying the POA algorithm, the accuracy increased to 97%. Metaheuristic algorithms, such as POA, have helped to increase the performance of the classifier by identifying the correct combination of features, which is the main reason for increasing accuracy.

## 4. Limitations and managerial implications

This research is based on XAI techniques, metaheuristic optimization algorithms, and enhancing the interpretability of ML models in providing reliable decisions for clinicians diagnosing PD. The proposed framework provides accurate predictions for PD classification using real-world data and helps clinicians better understand the cause and nature of the disease. However, it also has some limitations. First, this research focuses on a specific ML model that provides effective solutions to real-world data. Advanced models, such as deep learning (DL) models, can identify more complex relationships through automated feature extraction, which can further improve the model’s predictive ability. The integration of AI frameworks into healthcare information systems can help neurologists and other clinicians make decisions, thereby enabling early and accurate diagnosis of PD. This can create opportunities for rapid and targeted interventions for patients, improving their quality of life. However, technical and clinical skills are essential to implement these advanced AI tools, which is why medical institutions need training programs so that medical staff can properly understand and use the AI system’s output. The proposed framework is developed and validated using real data from the PPMI database, which ensures its applicability in real clinical environments. The technique, developed using MRI scans and patient data, mirrors real-world situations. However, to further confirm the effectiveness of the proposed algorithms, the authors have initiated collaboration with leading hospitals through their affiliated universities. Our framework can accurately map the progress of PD, but despite the reliability of XAI, it needs a more user-friendly interface. Early and accurate diagnosis of PD is important, as delay makes the condition difficult to manage. Future work should develop a large language model to improve the diagnostic model, which is necessary to ensure better clinical validation. Hospital collaboration will play an important role in this effort.

## 5. Conclusion and future directions

In the proposed study, an XAI-integrated framework is developed and a metaheuristic optimization algorithm, i.e., OA, is applied to the ML classification model for the early diagnosis of PD. This framework improves disease prognosis as well as provides detailed insights into predictions through XAI techniques and accurately identifies important features behind those decisions, helping clinicians to develop effective diagnosis plans. The study developed an FS-based decision support system using features of voice signals from PD patients and healthy individuals. The main objective was to increase the performance and accuracy of the model by reducing the number of features. The LightGBM model achieved 95% accuracy. XAI models were integrated into LightGBM to provide interpretation of predictions, where features such as difference entropy and joint entropy were identified as important, facilitating interpretation by clinicians. In addition, the POA metaheuristic algorithm was applied for FS, which improved the accuracy of the model to 97%, showing remarkable performance. This study demonstrates the potential of XAI in the diagnosis and management of PD and highlights the training needs to enable medical professionals to effectively use AI insights to improve patient outcomes. To enhance the effectiveness of the proposed PD diagnostic tool, future research may focus on applying advanced AI technologies such as DL and reinforcement learning and using multi-modal neuroimaging datasets. In addition, future efforts may focus on incorporating more biomarkers and modifying XAI techniques to develop improved and personalized solutions for PD management.

## Author contributions

**Conceptualization:** Proloy Kumar Mondal, Haewon Byeon.

**Data curation:** Proloy Kumar Mondal.

**Formal analysis:** Proloy Kumar Mondal.

**Funding acquisition:** Haewon Byeon.

**Investigation:** Proloy Kumar Mondal, Haewon Byeon.

**Methodology:** Proloy Kumar Mondal, Haewon Byeon.

**Project administration:** Haewon Byeon.

**Resources:** Haewon Byeon.

**Software:** Proloy Kumar Mondal.

**Supervision:** Haewon Byeon.

**Validation:** Proloy Kumar Mondal, Haewon Byeon.

**Visualization:** Proloy Kumar Mondal, Haewon Byeon.

**Writing – original draft:** Proloy Kumar Mondal.

**Writing – review & editing:** Proloy Kumar Mondal, Haewon Byeon.

## References

[1] Das R. A comparison of multiple classification methods for diagnosis of Parkinson disease. Expert Syst Appl. 2010;37:1568–72.

[2] Eunus SI Rokoni S Armisha MD. Hybrid 3D CNN-LSTM model for rs-fMRI-based Parkinson’s prediction. J Indep Stud Res Comput. 2025;23:8–15.

[3] Karapinar Senturk Z. Early diagnosis of Parkinson’s disease using machine learning algorithms. Med Hypotheses. 2020;138:109603. 32028195 10.1016/j.mehy.2020.109603

[4] Amato F Rechichi I Borzì L Olmo G. Sleep quality through vocal analysis: a telemedicine application. In: 2022 IEEE International Conference on Pervasive Computing and Communications Workshops and other Affiliated Events (PerCom Workshops). IEEE; 2022:706–11.

[5] Pinto S Ozsancak C Tripoliti E Thobois S Limousin-Dowsey P Auzou P. Treatments for dysarthria in Parkinson’s disease. Lancet Neurol. 2004;3:547–56. 15324723 10.1016/S1474-4422(04)00854-3

[6] Pozzi NG Isaias IU. Adaptive deep brain stimulation: retuning Parkinson’s disease. Handb Clin Neurol. 2022;184:273–84. 35034741 10.1016/B978-0-12-819410-2.00015-1

[7] Moradi S Tapak L Afshar S. Identification of novel noninvasive diagnostics biomarkers in the Parkinson’s diseases and improving the disease classification using support vector machine. Biomed Res Int. 2022;2022:1–8. 10.1155/2022/5009892 PMC8941533 35342758

[8] Cordella F Paffi A Pallotti A. Classification-based screening of Parkinson’s disease patients through voice signal. In: 2021 IEEE International Symposium on Medical Measurements and Applications (MeMeA). IEEE; 2021:1–6.

[9] Ali L Chakraborty C He Z Cao W Imrana Y Rodrigues JJPC. A novel sample and feature dependent ensemble approach for Parkinson’s disease detection. Neural Comput Appl. 2022;35:15997–6010.

[10] Huang F Xu H Shen T Jin L. Recognition of Parkinson’s disease based on residual neural network and voice diagnosis. In: 2021 IEEE 5th Information Technology, Networking, Electronic and Automation Control Conference (ITNEC). vol. 5. IEEE; 2021:381–6.

[11] Jaeger H Trivedi D Stadtschnitzer M. Mobile device voice recordings at King’s College London (MDVR-KCL) from both early and advanced Parkinson’s disease patients and healthy controls. Zenodo. 2019.

[12] Wodzinski M Skalski A Hemmerling D Orozco-Arroyave JR Nöth E. Deep learning approach to Parkinson’s disease detection using voice recordings and convolutional neural network dedicated to image classification. In: 2019 41st Annual International Conference of the IEEE Engineering in Medicine and Biology Society (EMBC). IEEE; 2019:717–20. 10.1109/EMBC.2019.8856972 31945997

[13] Wroge TJ. Parkinson’s disease diagnosis using machine learning and voice. In: 2018 IEEE Signal Processing in Medicine and Biology Symposium (SPMB). IEEE; 2018:1–7.

[14] Ashour AS El-Attar A Dey N Abd El-Kader H Abd El-Naby MM. Long short term memory based patient-dependent model for FOG detection in Parkinson’s disease. Pattern Recognit Lett. 2020;131:23–9.

[15] Sharma S Moon CS Khogali A. Biomarkers in Parkinson’s disease (recent update). Neurochem Int. 2013;63:201–29. 23791710 10.1016/j.neuint.2013.06.005

[16] Prashanth R Roy SD Mandal PK Ghosh S. High-accuracy detection of early Parkinson’s disease through multimodal features and machine learning. Int J Med Inf. 2016;90:13–21. 10.1016/j.ijmedinf.2016.03.001 27103193

[17] Wang W Lee J Harrou F Sun Y. Early detection of Parkinson’s disease using deep learning and machine learning. IEEE Access. 2020;8:147635–46.

[18] Alkhatib R Diab MO Corbier C El Badaoui M. Machine learning algorithm for gait analysis and classification on early detection of Parkinson. IEEE Sens. Lett. 2020;4:1–4.35582432

[19] Ricciardi C. Machine learning can detect the presence of Mild cognitive impairment in patients affected by Parkinson’s Disease. In: 2020 IEEE International Symposium on Medical Measurements and Applications (MeMeA). IEEE; 2020:1–6.

[20] Haq AU Li JP Memon MH. Feature selection based on l1-norm support vector machine and effective recognition system for Parkinson’s disease using voice recordings. IEEE Access. 2019;7:37718–34.

[21] Mei J Desrosiers C Frasnelli J. Machine learning for the diagnosis of Parkinson’s disease: a review of literature. Front Aging Neurosci. 2021;13:633752. 34025389 10.3389/fnagi.2021.633752 PMC8134676

[22] Gunduz H. Deep learning-based Parkinson’s disease classification using vocal feature sets. IEEE Access. 2019;7:115540–51.

[23] uci/parkinsons | Workspace. data.world https://data.world/uci/parkinsons/workspace/project-summary

[24] Luque A Carrasco A Martín A de Las Heras A. The impact of class imbalance in classification performance metrics based on the binary confusion matrix. Pattern Recognit. 2019;91:216–31.

[25] Ke G. Lightgbm: a highly efficient gradient boosting decision tree. Adv Neural Inf Process Syst. 2017;30:3146–54.

[26] Remeseiro B Bolon-Canedo V. A review of feature selection methods in medical applications. Comput Biol Med. 2019;112:103375. 31382212 10.1016/j.compbiomed.2019.103375

[27] Jain D Singh V. Feature selection and classification systems for chronic disease prediction: a review. Egypt Inf J. 2018;19:179–89.

[28] Liu H Yu L. Toward integrating feature selection algorithms for classification and clustering. IEEE Trans Knowl Data Eng. 2005;17:491–502.

[29] Dokeroglu T Sevinc E Kucukyilmaz T Cosar A. A survey on new generation metaheuristic algorithms. Comput Ind Eng. 2019;137:106040.

[30] Deniz A Kiziloz HE Dokeroglu T Cosar A. Robust multiobjective evolutionary feature subset selection algorithm for binary classification using machine learning techniques. Neurocomputing. 2017;241:128–46.

[31] Dehghani M Trojano-Alvarado E Zeidabadi FA Hubálovský Š Trojovský P. Pelican optimization algorithm: a novel nature-inspired algorithm for engineering applications. Sensors. 2022;22:855. 35161600 10.3390/s22030855 PMC8838090

[32] Priyadharshini S. A Comprehensive framework for Parkinson’s disease diagnosis using explainable artificial intelligence empowered machine learning techniques. Alex Eng J. 2024;107:568–82.

[33] Lundberg S. A unified approach to interpreting model predictions. ArXiv Prepr. 2017:ArXiv170507874.

[34] Ribeiro M. T. Singh S. & Guestrin C. “Why should i trust you?”: Explaining the predictions of any classifier. In: Proceedings of the 22nd ACM SIGKDD International Conference on Knowledge Discovery and Data Mining, ACM, San Francisco California USA, 2016:1135–44.

